# WhiB4 Is Required for the Reactivation of Persistent Infection of Mycobacterium marinum in Zebrafish

**DOI:** 10.1128/spectrum.00443-21

**Published:** 2022-03-10

**Authors:** Chen Lin, Yuting Tang, Yuchen Wang, Junli Zhang, Yeyu Li, Shuqin Xu, Bin Xia, Qiran Zhai, Yao Li, Lu Zhang, Jun Liu

**Affiliations:** a State Key Laboratory of Genetic Engineering, School of Life Science, Institute of Genetics, Fudan University, Shanghai, China; b Department of Microbiology, School of Life Science, Fudan University, Shanghai, China; c Shanghai Engineering Research Center of Industrial Microorganisms, Shanghai, China; d Department of Molecular Genetics, University of Toronto, Toronto, Ontario, Canada; e Beijing Nuclear Magnetic Resonance Center, College of Chemistry and Molecular Engineering, School of Life Sciences, Peking University, Beijing, China; Indian Institute of Science Bangalore

**Keywords:** WhiB4, latency, reactivation, tuberculosis, zebrafish

## Abstract

Granulomas are the pathological hallmark of tuberculosis (TB). In individuals with latent TB infection, Mycobacterium tuberculosis cells reside within granulomas in a nonreplicating dormant state, and a portion of them will develop active TB. Little is known on the bacterial mechanisms/factors involved in this process. In this study, we found that WhiB4, an oxygen sensor and a transcription factor, plays a critical role in disease progression and reactivation of Mycobacterium marinum (M. marinum) infection in zebrafish. We show that the *whiB4*::Tn mutant of M. marinum caused persistent infection in adult zebrafish, which is characterized by the lower but stable bacterial loads, constant number of nonnecrotized granulomas in fewer organs, and reduced inflammation compared to those of zebrafish infected with the wild-type bacteria or the complemented strain. The mutant bacteria in zebrafish were also less responsive to antibiotic treatments. Moreover, the *whiB4*::Tn mutant was defective in resuscitation from hypoxia-induced dormancy and the DosR regulon was dysregulated in the mutant. Taken together, our results suggest that WhiB4 is a major driver of reactivation from persistent infection.

**IMPORTANCE** About one-quarter of the world’s population has latent TB infection, and 5 to 10% of those individuals will fall ill with TB. Our finding suggests that WhiB4 is an attractive target for the development of novel therapeutics, which may help to prevent the reactivation of latent infection, thereby reducing the incidences of active TB.

## INTRODUCTION

Tuberculosis (TB), caused by Mycobacterium tuberculosis, is a leading infectious disease that caused 1.4 million deaths and 10.0 million new cases in 2019. In addition, an estimated 1.7 billion individuals are asymptomatically infected with M. tuberculosis, which is called latent TB infection (LTBI) (1). LTBI represents a huge disease reservoir since 5% to 10% of infected people will develop active disease. Currently, the reactivation of LTBI represents one of the major challenges to the effective control of the disease, as present vaccination strategies do not protect against this phase of infection and there are no antibiotics specifically targeting the latent organisms. Mathematical modeling showed that new interventions aimed at people with LTBI would be the most effective means for reducing the incidents and mortality associated with TB (2).

In individuals with LTBI, M. tuberculosis bacilli are thought to reside within the granulomas, which are stratified structures consisting of macrophages, giant cells, and foamy macrophages, surrounded by a lymphocyte-rich marginal zone and a fibrous capsule of collagen and other extracellular matrix proteins (3, 4). The development of granulomas and their subsequent degeneration and necrosis are the hallmark of the host response to infection with M. tuberculosis (5), which are complex processes and are likely dependent on the interplay between multiple bacterial and host factors (6, 7). Granuloma is thought to benefit the host by containing the bacteria but may also benefit the bacteria by providing a niche for growth and spread (8). The center of the human granuloma is considered to be a hypoxic environment due to the lack of endothelial and blood vessel markers (3, 4). Within granulomas, M. tuberculosis cells are thought to survive in a nonreplicating “dormant” state in response to the lack of oxygen and nutrients; as such, they are unresponsive to currently available antibiotics that typically target actively growing cells, which has been termed “phenotypic drug resistance” or “drug tolerance” (9–12). Hypoxia-induced dormancy has been studied extensively, and the DosR regulon has been identified as a key factor for initiation of dormancy *in vitro* (13–15) and for persistence *in vivo* (16).

During reactivation of LTBI, the dormant bacteria are believed to resuscitate and resume normal growth and metabolism. However, little is known on the mechanisms of reactivation (17). Based on the observation that reactivation of LTBI in humans occurs most frequently in the upper lobes of the lung, which is the most oxygenated region of the body (18), a few recent studies have used reaeration of hypoxic cultures for *in vitro* modeling of reactivation or resuscitation, and several regulatory proteins, such as transcription factor ClgR (19, 20) and sigma factors SigH (21) and SigE (22, 23), were identified. However, they have not been studied in *in vivo* models.

WhiB4 is a member of the WhiB superfamily of transcription factors that are conserved in mycobacterial species, including M. tuberculosis and Mycobacterium marinum (24). WhiB4 is an oxygen sensor and contains the highly conserved Cys-X14-22-Cys-X2-Cys-X5-Cys motif, which forms a [4Fe-4S] cluster in the holoenzyme (25, 26). Upon exposure to O_2_, the [4Fe-4S] cluster is rapidly (within minutes) oxidized, and the oxidized form has stronger DNA binding activity (26). The response of WhiB4 to oxygen exposure occurred much more rapidly than that of other WhiB proteins (e.g., WhiB1 and WhiB3), making it a primary oxygen sensor (26–28). Previously, we showed that WhiB4 is required for the virulence of M. marinum in zebrafish (29). However, the underlying mechanism is unknown.

In this study, we show that infection of adult zebrafish with the *whiB4*::Tn mutant of M. marinum resulted in persistent infection, which is characterized by the stable bacterial loads, constant number of nonnecrotized granulomas in fewer organs, and reduced inflammation compared to those of zebrafish infected with the wild-type (WT) bacteria or the complemented strain. The mutant bacteria in zebrafish were also less responsive to antibiotic treatments. We show that the *whiB4*::Tn mutant was defective in resuscitation from hypoxia-induced dormancy and that the DosR regulon was dysregulated in the mutant. Taken together, our results suggest that WhiB4 is a bacterial factor that is critically required for the M. marinum-induced pathogenesis and disease progression in zebrafish. The zebrafish-M. marinum *whiB4*::Tn infection provides an attractive animal model for LTBI, which could be useful for studies of host factors affecting disease progression, as well as drug screening and vaccine development targeting the dormant bacteria.

## RESULTS

### M. marinum *whiB4*::Tn mutant induces partially necrotic and stable granulomas.

**FIG 1:**
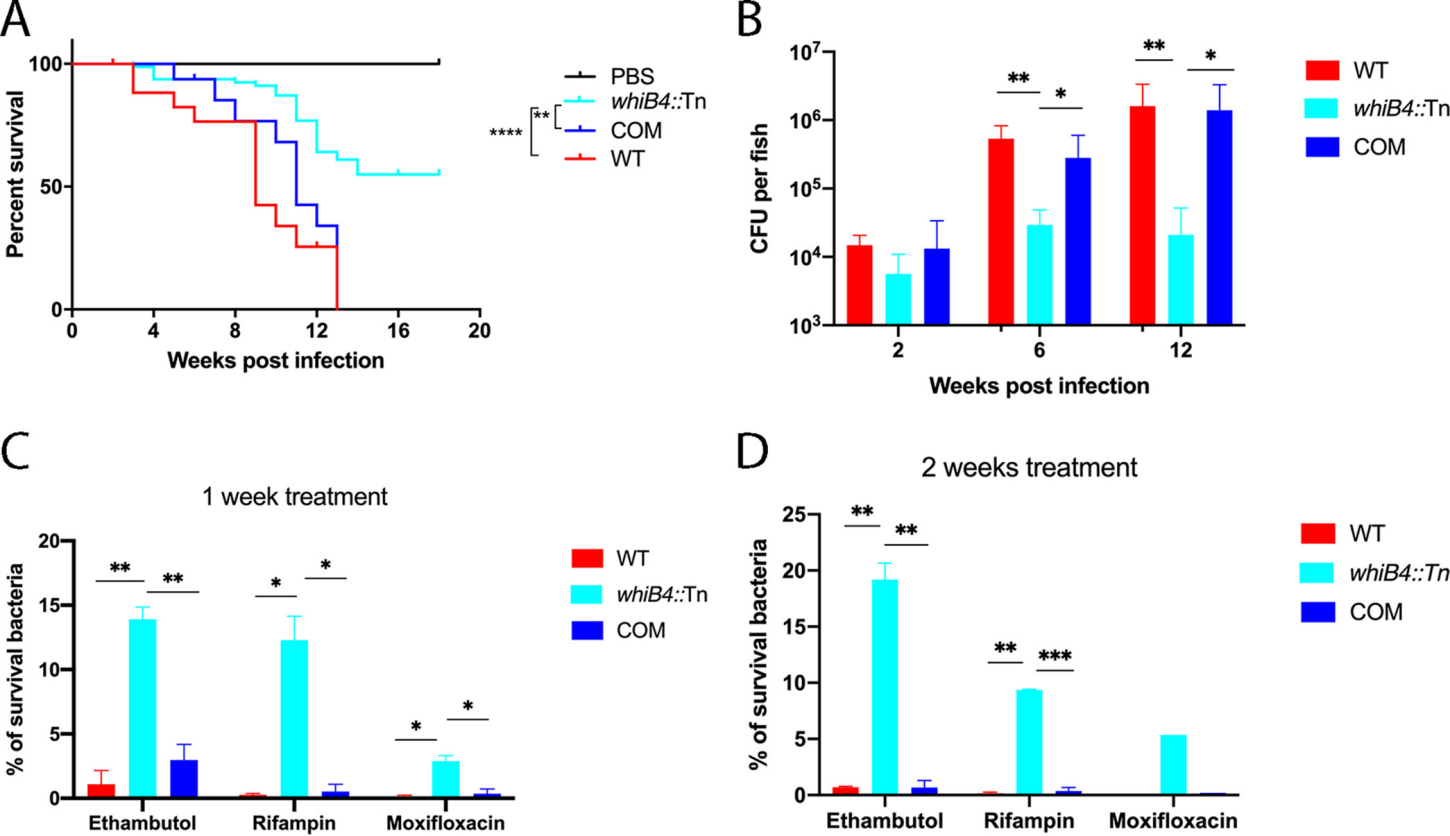
(A and B) The w*hiB4*::Tn mutant is attenuated in zebrafish. (A) Adult zebrafish (*n* = 20) were infected with the indicated strains at 10^4^ CFU bacteria per fish and were monitored for mortalities over an 18-week period. The survival curves were plotted using the Kaplan-Meier method and differences between curves were analyzed using the log-rank test. **, *P < *0.01; ****, *P* < 0.0001. (B) In parallel experiment, zebrafish (*n* = 20) were infected with the same strains at 10^4^ CFU bacteria per fish, and at indicated time points (2, 6, and 12 weeks) postinfection, 3 live fish per group were sacrificed and bacterial burdens were determined. Data are plotted as mean ± standard error of the mean (SEM). Unpaired Student’s *t* test: *, *P < *0.05; **, *P < *0.01. (C and D) *whiB4*::Tn in infected zebrafish is more tolerant to antibiotic killing than WT. In a separate experiment, zebrafish were infected with the indicated strains at a dose of 10^4^ CFU/fish. At 2 weeks postinfection, animals (*n* = 5) were treated with ethambutol (1 mM), rifampicin (0.4 mM), or moxifloxacin (0.3 mM) for 1 or 2 weeks, and they were then sacrificed and bacterial burdens were determined. Data are plotted as mean ± standard deviation (SD). Unpaired Student’s *t* test: *, *P < *0.05; **, *P < *0.01. COM, the complemented strain.

Previously, we observed that at 3 to 4 weeks postinfection, well-organized granulomas appeared in adult zebrafish intraperitoneally infected with the *whiB4*::Tn mutant. In contrast, in zebrafish infected with the wild-type (WT) M. marinum, the bacteria had spread extensively and there were no organized granulomas observed at this stage of infection (29). To examine this more closely, we repeated this experiment but extended the period of the experiment to 18 weeks. Consistent with our previous finding, zebrafish infected with the *whiB4*::Tn mutant exhibited prolonged survival compared to that of those infected with WT or the complemented strain, indicating that the *whiB4*::Tn mutant is attenuated in zebrafish (Fig. 1A).

**FIG 2:**
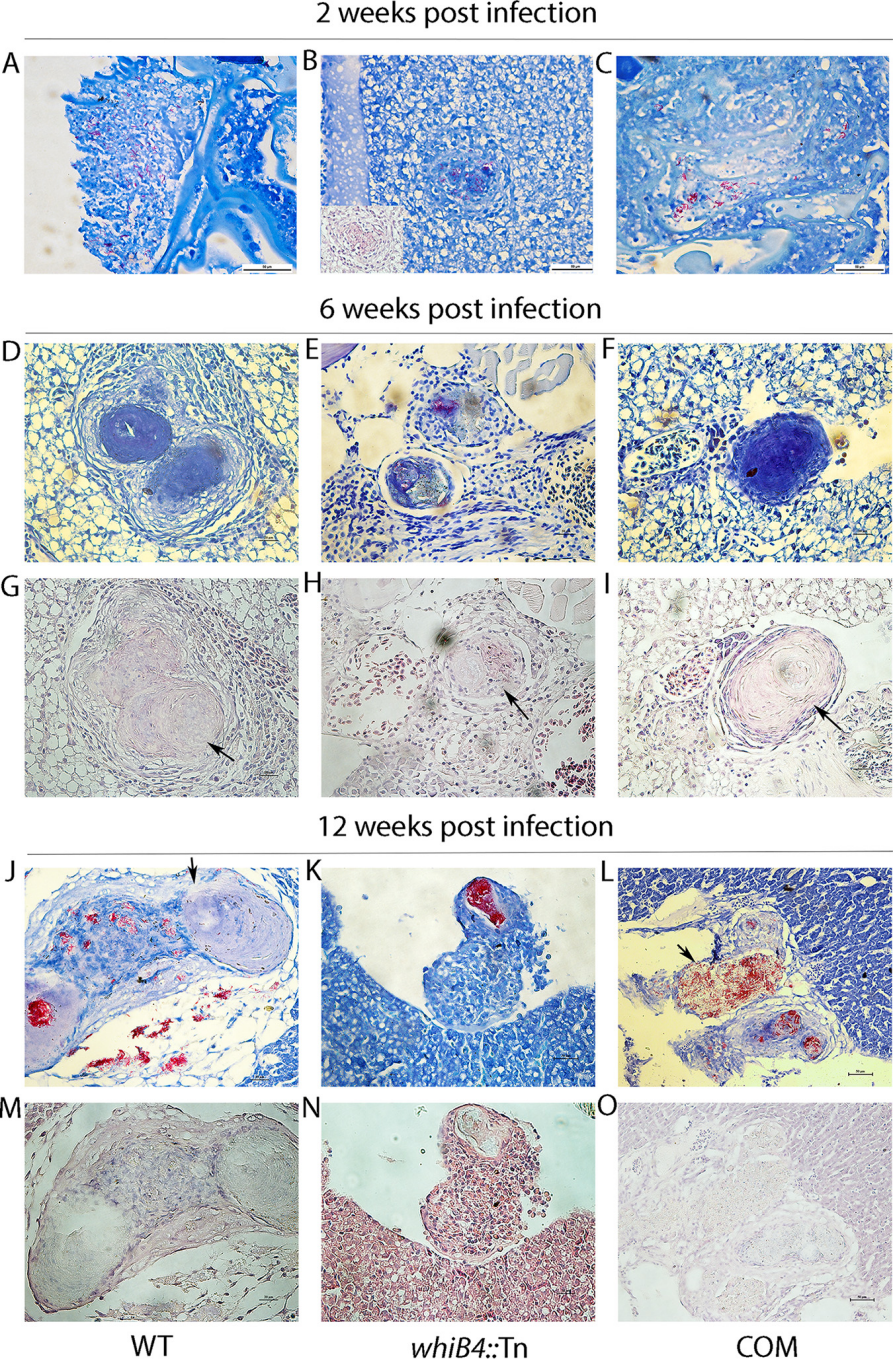
The *whiB4*::Tn mutant induces partially necrotic and stable granulomas. Zebrafish were infected with the indicated strains as described in Fig. 1A and B. At different time points (2, 6, and 12 weeks) postinfection, animals (*n* = 3) were sacrificed and subjected to histological analysis. Ziehl-Neelsen acid fast staining: panels A to F and J to L. Hematoxylin and eosin (H&E) staining: panel B (*inset*) and panels G to I and M to O. COM, the complemented strain.

We also performed histological analysis at more time points (2, 6, and 12 weeks postinfection). Live fish (*n* = 3) per group at each time point were sacrificed, and granulomas were examined. At 2 weeks postinfection, loosely organized granulomas were detected in animals infected with *whiB4*::Tn (Fig. 2B, inset). In contrast, in zebrafish infected with the WT or the complemented strain, the bacteria disseminated throughout the organs and no granuloma-like structures were detected (Fig. 2A and C).

At 6 weeks postinfection, approximately equal numbers of well-organized granulomas were detected in all animals, including those infected with WT, *whiB4*::Tn, or the complemented strain (Fig. 2D to F). However, the granulomas formed in zebrafish infected with *whiB4*::Tn appeared only partially necrotic (Fig. 2H, arrow), where cellular remnants were seen inside the granulomas. This is in contrast to the fully necrotized granulomas observed in zebrafish infected with WT or the complemented strain (Fig. 2G and I). At this stage, bacteria were predominantly found inside granulomas in all animals, with few free bacteria detected.

At 12 weeks postinfection, the number of granulomas (2 to 3 granulomas per sample) detected in zebrafish infected with *whiB4*::Tn was similar to that at 6 weeks postinfection. However, in zebrafish infected with WT or the complemented strain, the number of granulomas increased dramatically, from 2 to 3 per sample at week 6 to on average 24 per sample at week 12 postinfection. In addition, the thin-walled and completely necrotized granulomas in animals infected with WT or the complemented strain were mostly broken down, leading to the spread of the bacteria throughout the body (Fig. 2J, L, M, and O). In contrast, in zebrafish infected with *whiB4*::Tn, the granulomas remained intact and the bacteria were detected only inside the granulomas and were not disseminated (Fig. 2K and N).

The pathological features of the animals correlated with the bacterial burden. In zebrafish infected with the WT or the complemented strain, the bacteria multiplied substantially over the 12-week period, reaching over 10^6^ CFU per fish, which was more than 2.0 log_10_ higher than the infection dose (10^4^ CFU) (Fig. 1B). In contrast, the number of granulomas infected with *whiB4*::Tn increased only slightly within the first 6 weeks and was stabilized from weeks 6 to 12 (Fig. 1B). Consistently, we did not observe the expansion of granuloma number in zebrafish infected with *whiB4*::Tn from weeks 6 to 12, as was observed in those infected with WT or the complemented strains.

Taken together, these results suggest that compared to the WT or the complemented strain, the *whiB4*::Tn mutant induced less necrotic and more stable granulomas in adult zebrafish, and consequently, the replication of the bacteria was controlled and did not lead to invasive and disseminated disease. It also appears that threshold of bacterial burden required to trigger the formation of granulomas is much higher (∼100-fold) for WT or the complemented strain than for the *whiB4*::Tn mutant, as similar numbers of granulomas were found in zebrafish infected with all three strains at week 6 postinfection but the bacterial load of the animals infected with WT or the complemented strain was ∼2.0 log_10_ higher than that of those infected with *whiB4*::Tn at this time point. The higher bacterial burden, combined with the necrotic nature of granulomas formed in animals infected with WT or the complemented strain, may lead to granuloma expansion and bacterial dissemination, as observed at week 12 postinfection.

### *whiB4*::Tn in infected zebrafish is more tolerant to antibiotic killing than WT.

The above finding also suggests that the *whiB4*::Tn mutant might be in a dormant state in zebrafish, resembling M. tuberculosis in human LTBI. To test this, we repeated the infection experiment of zebrafish with WT, *whiB4*::Tn, and the complemented strain. Two weeks postinfection, ethambutol (1.0 mM), rifampicin (0.4 mM), or moxifloxacin (0.3 mM) was added to the tanks of the animals (*n* = 6 per group), and the tank water was changed daily, each time with the antibiotic solution added. Equal numbers of animals that were infected with the three M. marinum strains but not treated with antibiotics were used as the control, respectively. The drug treatment lasted for 1 or 2 weeks; at each period, the zebrafish were sacrificed and bacterial burden was determined.

Interestingly, while all three drugs were highly effective in killing the WT or the complemented strain in infected zebrafish, they were much less effective in killing the *whiB4*::Tn mutant (Fig. 1C and D). For example, for WT and the complemented strain, nearly all bacteria (>99%) were killed by rifampicin and moxifloxacin, whereas about 10% of *whiB4*::Tn cells survived the killing by rifampicin and 3 to 5% of *whiB4*::Tn cells survived the killing by moxifloxacin (Fig. 1C and D). Since the MICs of these drugs against *whiB4*::Tn were the same as those against WT or the complemented strain, and the *in vitro* drug tolerance assay did not show any differences among the three strains (Fig. S1), these results suggest that the *whiB4*::Tn mutant in zebrafish was more tolerant against antibiotic killing. Interestingly, a recent *in vitro* study found that disruption of *whiB4* in M. tuberculosis resulted in enhanced tolerance to the combined treatment of amoxicillin and clavulanate (30). However, the mechanism appears to be different from what we have observed here for the *whiB4*::Tn mutant of M. marinum *in vivo*.

Taken together, the above results are consistent with the notion that the *whiB4*::Tn mutant induced granuloma formation and entered dormant state at an early stage (2 weeks postinfection), whereas the WT and the complemented strain did not induce granuloma formation until a later time point (6 weeks postinfection) and were susceptible for antibiotic killing at 2 weeks postinfection.

### *whiB4*::Tn inhibits stimulation of host signaling pathways.

Our data thus far are consistent with the notion that nonnecrotized granulomas led to the effective control of the *whiB4*::Tn mutant and, consequently, the infected zebrafish does not develop into invasive and disseminated disease. To gain insight into the host factors involved in this process, we performed transcriptome sequencing (RNA-seq) analysis of kidneys isolated from zebrafish infected with *whiB4*::Tn or WT at 2 weeks postinfection. We chose kidneys for this analysis since at this time point there were no bacteria detected in this organ in either group of the animals.

A total of 4,181 differentially expressed genes (DEGs; fold difference of >1.5, *P < *0.05) were detected between zebrafish infected with *whiB4*::Tn and those infected with WT, including 3,093 downregulated and 1,088 upregulated genes (Table S1).

**FIG 3:**
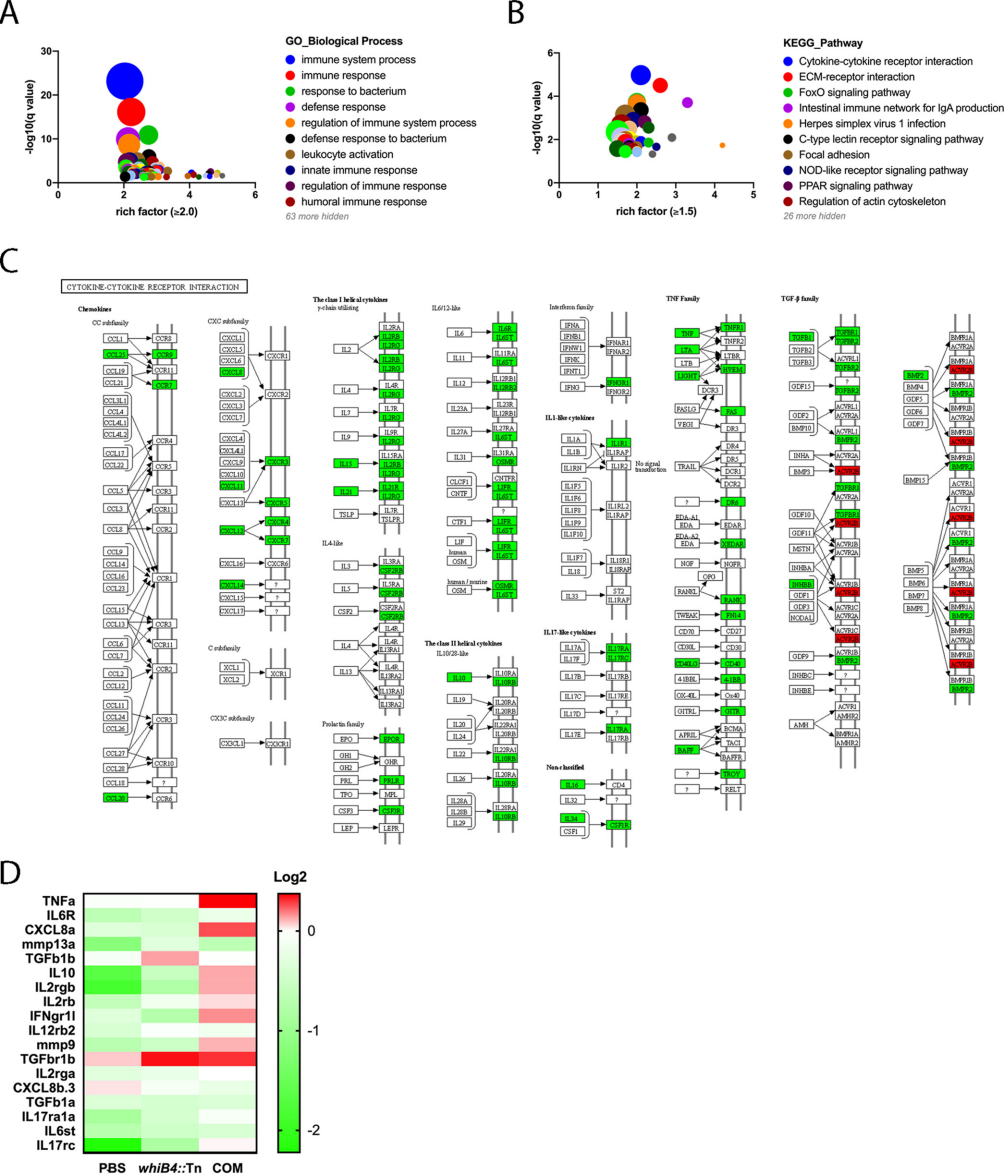
The *whiB4*::Tn mutant induces lower levels of host immune response than the WT strain. RNA-seq analysis of kidneys isolated from zebrafish infected with *whiB4*::Tn or WT at 2 weeks postinfection was performed. The identified DEGs were subjected to GO analysis (A) and KEGG pathway analysis (B). Each circle represents a biological process or pathway, and the size of the circle is proportional to the number of genes detected. (C) Map DEGs involved in the cytokine-cytokine receptor interaction pathway. Green, downregulated; red, upregulated. (D) RT-qPCR validation of selected DEGs. Heatmap of 18 DEGs analyzed by RT-qPCR. The mRNA levels were normalized against WT and 18S rRNA was used as an internal control. COM, the complemented strain.

We performed gene ontology (GO) analysis of these DEGs and found that the largest group of enriched DEGs (*n* = 345) belongs to the “immune system process” category (Fig. 3A, Table S2). About 90% of these genes (*n* = 311) were downregulated in zebrafish infected with *whiB4*::Tn compared to those infected with WT M. marinum. The KEGG pathway analysis also revealed that the cytokine-cytokine receptor interaction is the major pathway that was enriched (Fig. 3B, Table S3). Among the 65 DEGs in this pathway, 64 were downregulated in zebrafish infected with *whiB4*::Tn compared to those infected with WT (Fig. 3C). These included proinflammatory cytokines and chemokines such as tumor necrosis factor (TNF) and C-X-C motif chemokine 11 (CXCL-11) and/or receptors such as tumor necrosis factor receptor 1 (TNFR1), CXCR3, interleukin 1 receptor type 1 (IL-1R1), IL-6R, and interleukin 6 cytokine family signal transducer (IL-6ST), as well as pleiotropic cytokines IL-15 and IL-21.

To validate the RNA-seq data, we selected 16 DEGs that were identified in the cytokine-cytokine receptor interaction pathway and 2 DEGs of matrix metallopeptidases to perform reverse transcriptase quantitative PCR (RT-qPCR) analysis (Fig. 3D). These 18 genes have been implicated in host immune response to M. tuberculosis infection (reviewed in reference 31). We also included kidneys from zebrafish infected with the complemented strain or the noninfected phosphate-buffered saline (PBS) control for comparison. The RT-qPCR results showed that the majority of these genes were expressed at lower levels in zebrafish infected with *whiB4*::Tn than in those infected with WT or the complemented strain, with an overall expression profile more closely resembling that of zebrafish that were not infected (the PBS control group) (Fig. 3D).

Other notable pathways that were significantly enriched include the NOD-like receptor signaling pathway that is responsible for detecting various pathogens and generating innate immune response and the MAPK signaling pathway that responds to proinflammatory stimuli. The majority of the DEGs in these two pathways were downregulated in zebrafish infected with *whiB4*::Tn (Fig. 3C, Table S3).

Taken together, these results suggest that the *whiB4*::Tn mutant induced less inflammation in zebrafish than the WT or the complemented strain.

### *whiB4*::Tn is defective in resuscitation from hypoxia-induced dormancy.

Thus far, we have demonstrated that zebrafish infected with *whiB4*::Tn resembled a latent infection and the bacteria were likely in a dormant state. To understand the underlying bacterial mechanisms, we examined the growth of *whiB4*::Tn under hypoxia using the Wayne model (12, 32). In this model, a sealed and slowly shaking culture is incubated over an extended period where the bacteria deplete available oxygen. The gradual depletion of oxygen leads to nonreplicating persistence states (NRP) with a concomitant shift in gene expression and metabolism. Under these conditions, M. marinum entered the NRP 1 state after 48 h incubation, characterized by a slight increase in turbidity without a corresponding increase in CFU. Full anaerobiosis was achieved after 132 h of incubation, as indicated by the complete decolorization of methylene blue, and the bacteria were in the NRP2 state (Fig. S2A). M. marinum resumed active growth after reaeration (Fig. S2B).

**FIG 4:**
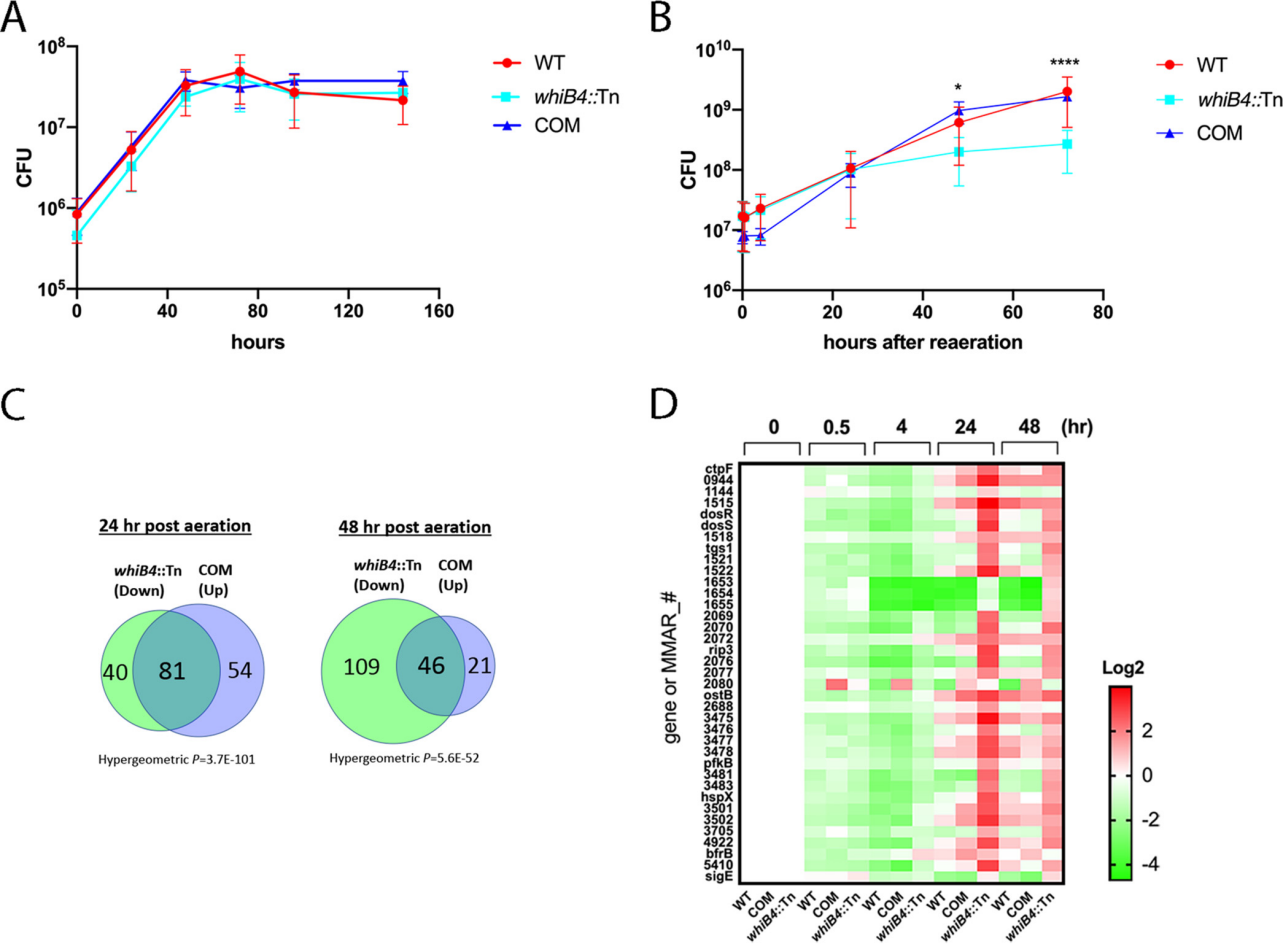
The *whiB4*::Tn mutant is defective in resuscitation from hypoxia-induced dormancy. (A) Growth of WT, *whiB4*::Tn, and the complemented strain (COM) under hypoxia. Data (mean ± SD) are from three biological replicates. There was no significant difference on the growth curves as analyzed by two-way analysis of variance (ANOVA). (B) Growth of hypoxic cultures of WT, *whiB4*::Tn, and the complemented strain (COM) after reaeration. Data (mean ± SD) are from three biological replicates. The CFU of *whiB4*::Tn was significantly lower than that of WT or the complemented strain at 48 and 72 h postaeration. *, *P < *0.05; ****, *P < *0.0001, two-way ANOVA. (C) Overlaps of DEGs in *whiB4*::Tn and the complemented strain (COM). Genes that were downregulated in *whiB4*::Tn relative to WT overlapped significantly with genes that were upregulated in the complemented strain relative to WT at 24 and 48 h postaeration. (D) Heatmap of the mRNA levels of the DosR regulon and *sigE*. The mRNA level of each gene was normalized against the value of the same gene at 0 h time point (7-day hypoxic cultures) and plotted as log_2_.

Compared to WT or the complemented strain, the *whiB4*::Tn mutant did not exhibit growth defect under hypoxic conditions (Fig. 4A). However, when w*hiB4*::Tn was resuscitated by reaeration after 7-day incubation under hypoxia, there was a defect on its ability to regrow (Fig. 4B). Since *whiB4*::Tn grows equally well as WT and the complemented strain under aerobic conditions (normoxia) (see Fig. S3) (29), this result suggests that *whiB4*::Tn is defective in recovery from hypoxia-induced dormancy.

### The DosR regulon is dysregulated in the *whiB4*::Tn mutant.

To gain insight into the molecular mechanisms underlying the defective recovery of *whiB4*::Tn from hypoxia, we grew WT and *whiB4*::Tn under hypoxia for 7 days using the Wayne model and then reaerated the cultures. At different time points thereafter (0, 0.5, 4, 24, 48 h), M. marinum cultures were collected and subjected to RNA-seq analysis. A total of 30 samples were collected (three biological replicates at each time point) and analyzed.

The RNA-seq reads show a high mapping ratio for all samples (>96%) (Table S4), indicating the overall high sequencing accuracy. We compared the RNA-seq data of *whiB4*::Tn and WT at each time point.

Twenty-four DEGs (>2-fold, *q *< 0.05) were detected between *whiB4*::Tn and WT after 7-day incubation under hypoxia (Table S5, zero time point postaeration), of which 22 were downregulated, including 5 genes of the *mce1* operon (*mce1A*–*1D* and *lprK*).

At the earliest time point after resuscitation (0.5 h), only 9 DEGs were detected between *whiB4*::Tn and WT. However, the number of DEGs increased to 28, 175, and 243 at 4, 24, and 48 h postaeration, respectively (Table S5). This corresponds to the growth pattern of *whiB4*::Tn and WT after reaeration, in which differential growth between *whiB4*::Tn and WT began to show at 24 h postaeration (Fig. 4B). We therefore focused on the DEGs of *whiB4*::Tn and WT at the 24- and 48-h time points. As expected, there is a large overlap (151 DEGs) between these two time points, accounting for 86.3% and 62.1% of DEGs detected at 24 and 48 h postaeration, respectively.

Of the 175 DEGs detected at 24 h postaeration, 121 were downregulated in *whiB4*::Tn compared to WT. Interestingly, 5 genes belonging to the DosR regulon (*dosS*, *pfkB*, *MMAR_2069*, *MMAR_2070*, *MMAR_2076*) were upregulated (Table S5). Consistently, these five genes were also upregulated in *whiB4*::Tn at 48 h postaeration. In addition, another 6 DosR regulon genes were also upregulated in *whiB4*::Tn at 48 h postaeration, including *ctpF*, *MMAR_1521*, *rip3*, *MMAR_3483*, *MMAR_3501*, and *MMAR_3705* (Table S5). Importantly, none of the DosR regulon genes was among the downregulated genes in *whiB4*::Tn at the same time points.

To determine if the observed phenotypes could be restored in the complemented strain, we performed additional RNA-seq experiments and compared the expression profile of the complemented strain with that of the WT. A number of DEGs were detected in the complemented strain at different time points, which were 71, 417, 229, 143, and 69 at times 0, 0.5, 4, 24, and 48 h postaeration, respectively (Table S6). This is likely due to the overexpression of *whiB4* in the complemented strain, as the *whiB4* was cloned into a multicopy plasmid pNBV1 for complementation (29).

Starting from the time point at 0.5 h postaeration, the majority of the DEGs in the complemented strain were upregulated. As expected, there were significant overlaps between DEGs that were upregulated in the complemented strain and those that were downregulated in the *whiB4*::Tn mutant (Fig. 4C). For example, at 24 h postaeration, 135 DEGs were upregulated in the complemented strain; 81 of them overlapped with the downregulated DEGs in the *whiB4*::Tn mutant. In contrast, there was no overlap between the upregulated DEGs of these two strains at this time point. Importantly, none of the DosR regulon genes was upregulated in the complemented strain compared to WT (Table S6). Similar results were found for cultures at 48 h postaeration.

To gain a better view, we plotted the relative mRNA levels of individual DosR regulon in WT, the complemented strain, and *whiB4*::Tn at different time points postaeration by normalizing against the values at the 0 h time point (7-day hypoxic cultures). The analysis shows that at 0.5 and 4 h postaeration, the majority of the DosR regulon genes were downregulated in WT and the complemented strain. In contrast, some of these genes were not downregulated and even showed increased expression in *whiB4*::Tn at the same time points (Fig. 4D). At 24 and 48 h postaeration, the majority of genes began to show increased expression in WT, the complemented strain, and *whiB4*::Tn; however, the levels of expression were generally higher in *whiB4*::Tn than in WT or the complemented strain (Fig. 4D). Taken together, these results indicate that the DosR regulon is dysregulated in *whiB4*::Tn, which could explain its growth defect during resuscitation from the hypoxia-induced dormancy (Fig. 4B).

Several regulatory genes were also upregulated in *whiB4*::Tn, including *sigE* (Fig. 4D), *MMAR_1569* which encodes a TetR family transcription factor, and *MMAR_1825* encoding a probable transcription factor (Table S5).

Another notable family of genes that were differentially expressed in *whiB4*::Tn is the *pe*/*ppe* family of genes. A total of 33 and 45 *pe*/*ppe* genes were downregulated in *whiB4*::Tn compared to WT at 24 and 48 h postaeration, respectively, and only 2 *pe*/*ppe* genes were upregulated in *whiB4*::Tn at these time points (Table S5). This is consistent with our previous finding that WhiB4 is a major regulator of the *pe/ppe* family genes and primarily acts as a positive regulator (29).

### WhiB4 binds the *dosS* gene in hypoxic culture upon aeration.

The above data suggest that WhiB4 may directly bind to members of the DosR regulon, thereby inhibiting their expression. To test this, we cloned the *whiB4* gene containing a C-terminal FLAG tag sequence into the expression vector pMV261. The resulting construct was transformed into the WT strain. The recombinant strain was grown under hypoxia using the same conditions described above for 7 days. At this time point and 0.5 h postaeration, the cultures were collected and subjected to chromatin immunoprecipitation sequencing (ChIP-seq) analysis. Separately, the recombinant strain was also grown under normoxia to log phase and the culture was collected and subjected to ChIP-seq analysis.

**FIG 5:**
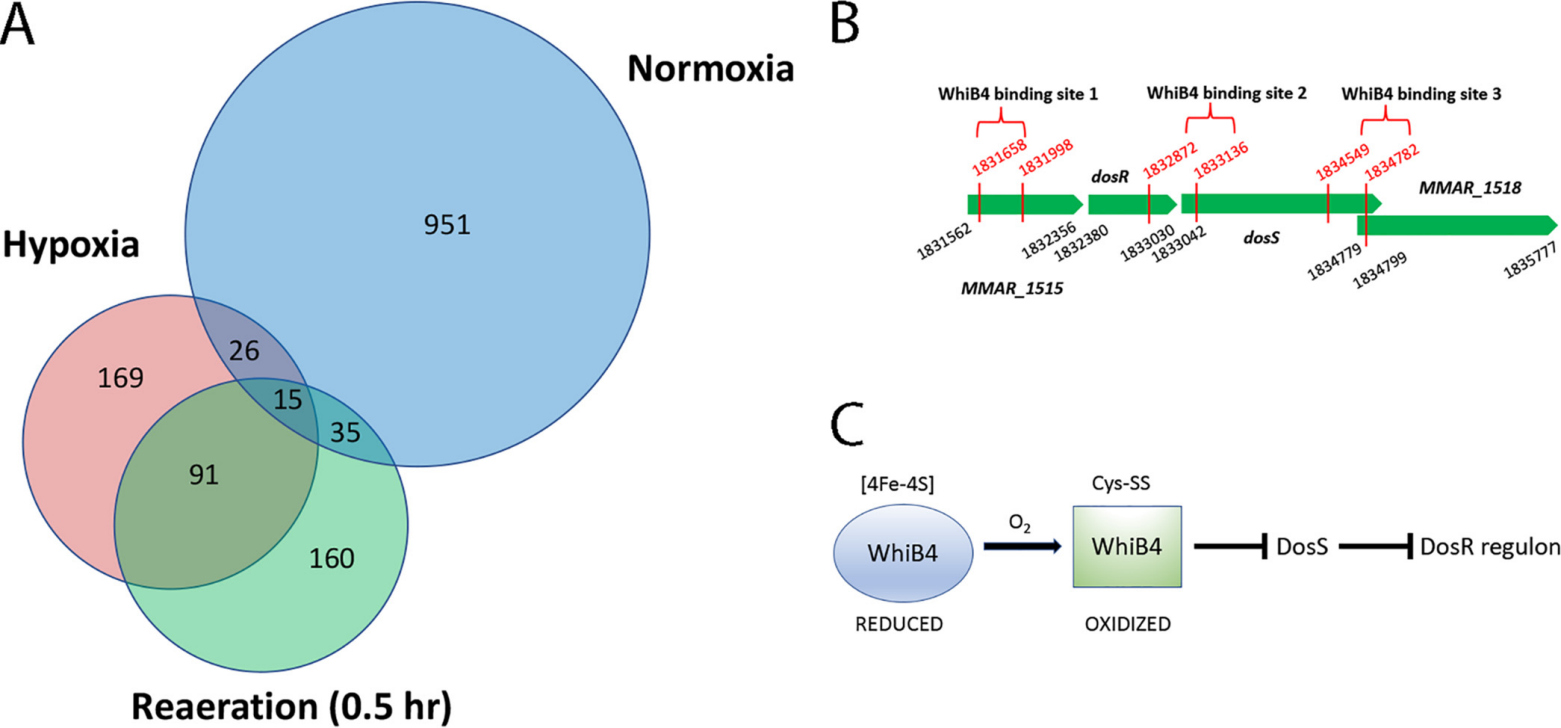
WhiB4 binds *dosS* in hypoxic culture upon aeration. (A) Overlap of the WhiB4 binding sites in bacteria grown under different conditions. (B) WhiB4 binding sites at the *dosR*-*dosS* locus. Binding sites 1 and 2 were detected in cultures at 0.5 h postaeration. Binding sites 1 and 3 were detected in hypoxic cultures without aeration. The numbers in black are the genome coordinates of the indicated genes, and the numbers in red indicate the binding site of WhiB4. (C) Model for the WhiB4-mediated regulation of the DosR regulon. Upon aeration of hypoxic cultures, WhiB4 binds directly to *dosS* and inhibits its expression, and consequently the expression of the DosR regulon is repressed.

A total of 302 unique binding sites of WhiB4 were identified in hypoxic cultures after 7 days of incubation (enrichment fold ≥ 1.5, *q* < 0.05) (Table S7). A similar number, 301, of the WhiB4 binding sites were identified in cultures at 0.5 h postaeration, and 106 of them overlapped with the binding sites in the hypoxic cultures (Fig. 5A). A total of 1,028 WhiB4 binding sites were identified in log-phase culture grown under normoxia, and only a small number of these sites (40 to 50) overlapped with the binding sites in hypoxic cultures (Table S7, Fig. 5A). These results suggest that WhiB4 binds distinct genes under hypoxia and normoxia. The higher number of binding sites under normoxia might be explained by the stronger DNA binding activity of the oxidized WhiB4 that is present under these conditions (26). Previously, ChIP-seq analysis of M. tuberculosis found that WhiB4 binds preferentially GC-rich sequences (33). We found a similar trend for WhiB4 in M. marinum under normoxia, although it is less apparent for the hypoxic cultures, presumably due to the fewer binding sites identified under this condition (Table S7, Fig. S4).

Interestingly, we found that WhiB4 directly bound to *dosS* in hypoxic culture at 0.5 h postaeration; WhiB4 bound a 265-bp fragment that includes 174 bp upstream of the start codon and 94 bp of the coding sequence of *dosS* (Table S7). The 174-bp upstream sequence also includes 154 bp of the *dosR* cording sequence at the 3′ end (Fig. 5B, binding site 2). This result suggests that WhiB4 likely blocks the promoter of *dosS*, thereby inhibiting its expression. This is consistent with the above observation that *dosS* was upregulated in the hypoxic cultures of *whiB4*::Tn at 24 and 48 h postaeration compared to the WT or the complemented strain at the same time points. Another WhiB4 binding site was also found within *MMAR_1515* (Fig. 5B, binding site 1), which is immediately upstream of *dosR* and *dosS.*

Two WhiB4-binding sites in the *dosR-dosS* locus were also found in hypoxic cultures without aeration (Table S7), including the same site within *MMAR_1515* (Fig. 5B, binding site 1) and a site that is located at the 3′ end of *dosS* and the overlapped 5′ of *MMAR_1518* (Fig. 5B, binding site 3). *MMAR_1515* and *MMAR_1518* likely form an operon with *dosR*-*dosS*, and they are also members of the DosR regulon.

In contrast, WhiB4 did not bind *dosS* or other genes in the *dosR-dosS* locus in log-phase cultures grown under normoxia (Table S7). Taken together, these results suggest a temporal control of the *dosS* expression by WhiB4, which is likely achieved by the oxygen concentration gradient encountered by the bacteria during resuscitation. Upon reaeration of the hypoxic cultures of WT (0.5 h postaeration), WhiB4 binds *dosS* and inhibits its expression, which leads to the downregulation of *dosS* and other members of the DosR regulon at later time points (24 and 48 h postaeration), allowing the resuscitation of the bacteria from the hypoxia-induced dormancy. In the *whiB4*:Tn mutant, the derepression of *dosS* and several other members of DosR regulon observed at 24 and 48 h postaeration could explain its growth defect at these time points (Fig. 4B and D).

## DISCUSSION

The majority of individuals infected with M. tuberculosis develop LTBI, which is characterized by immune-mediated disease control and lack of clinical symptoms. However, the control is eventually lost in a portion of these individuals, leading to active TB cases. Little is known on the bacterial mechanisms/factors involved in this process. In this study, we show that WhiB4 plays a critical role in disease progression and reactivation of M. marinum infection in zebrafish. This conclusion is supported by several observations. First, the *whiB4*::Tn mutant caused persistent infection in zebrafish which resembles latent infection, as evidenced by the low and constant bacterial load during the course of the infection and the attenuation phenotype. This suggests a successful control of bacterial multiplication by the host. Second, the granulomas induced by *whiB4*::Tn infection were stable and only partially necrotizing, which prevented the dissemination of the bacteria. Third, the *whiB4*::Tn bacilli appeared to be in a dormant state in the host, as they were less responsive to antibiotic treatment than the WT. Moreover, we found that *whiB4*::Tn was defective in resuscitation from hypoxia-induced dormancy, likely due to the dysregulation of the DosR regulon in the mutant. Taken together, our study provides new insight into the bacterial factors and host mechanisms underlying LTBI. Understanding the mechanisms of host-pathogen interactions in LTBI and its reactivation is necessary for the development of novel interventions targeting LTBI.

While the risk of LTBI reactivation is increased by factors that compromise the host immune system, such as HIV infection (34), diabetes (35), and glucocorticoid treatment (36), our results suggest that excessive inflammation may actually drive the disease progression, leading to invasive and disseminated disease. This was seen in zebrafish infected with the WT strain of M. marinum. Compared to those infected with the *whiB4*::Tn mutant, zebrafish infected with the WT bacteria exhibited higher levels of proinflammatory immune responses at the early time of infection (2 weeks postinfection). This heightened inflammation likely persists during the course of infection and contributes to the granuloma necrotic breakdown, thereby directly supporting bacterial dissemination and multiplication. Consequently, in zebrafish infected with WT or the complemented strain, the bacterial burden was significantly increased at later time points (Fig. 1B). Moreover, the expression of matrix metalloproteinases MMP-9 and MMP-13a was significantly upregulated in zebrafish infected with the WT M. marinum compared to that in zebrafish infected with the *whiB4*::Tn mutant (Fig. 3D). The matrix metalloproteinases are major contributors to the granuloma tissue remodeling owing to their abilities to degrade components of the extracellular matrix (ECM) such as collagen and proteoglycans (37). In human TB, several matrix metalloproteinases (MMPs), including MMP-1, -2, -8, -9, and -14, are markedly upregulated in expression in granulomas, and it was suggested that the upregulation of MMPs eventually leads to collagen destruction and granulomas necrosis (38–42). In zebrafish infected with the *whiB4*::Tn mutant, the overly vigorous inflammation and elevated expression of MMPs were tempered, and consequently, the granulomas remained intact and bacterial growth controlled, leading to persistent infection that resembles latent infection.

The reduced inflammation in zebrafish infected with *whiB4*::Tn is unlikely due to difference in bacterial load, since at 2 weeks postinfection, which was the time point at which the immune response was analyzed, there was no significant difference on the bacterial burden between animals infected with WT and those infected with *whiB4*::Tn (Fig. 1B). Instead, the reduced inflammation might be caused by the decreased expression of the *pe/ppe* family genes in *whiB4*::Tn (Table S5). Accumulated evidence suggests that PE/PPE family proteins play a critical role in host-pathogen interactions, and many PE/PPE proteins have been shown to modulate host immune response (43, 44). Consistent with our previous finding that WhiB4 positively regulates the *pe/ppe* family genes (29), we observed the downregulation of multiple *pe/ppe* genes in *whiB4*::Tn, which may contribute to the reduced inflammation observed in zebrafish infected with the mutant.

Our results suggest that WhiB4 is a major driver of reactivation from persistent infection. WhiB4 may achieve this role by inhibiting the expression of *dosS*, thereby shutting down the expression of DosR regulon during the resuscitation from dormancy (Fig. 5C). The DosR regulon of M. tuberculosis consists of 48 genes, and their expressions are controlled by a two-component regulatory system, the sensor kinase DosS and the response regulator DosR (13). The kinase function of DosS is activated by gaseous signal, including hypoxia, resulting in the induction of the 48 genes in the DosR regulon. However, upon exit from hypoxia-induced dormancy, the DosR regulon was downregulated in M. marinum (Fig. 4D), as was demonstrated in M. tuberculosis (23). Our results suggest that WhiB4 is the primary factor responsible for the downregulation of the DosR regulon under these conditions. The ChIP-seq analysis showed that WhiB4 directly bound the promoter region of *dosS* in hypoxic cultures upon aeration, which is expected to shut down the expression of the DosR regulon. Consistently, in the hypoxic cultures of *whiB4*::Tn mutant upon aeration, the *dosS* expression level remained abnormally high, resulting in the dysregulation of other members of the DosR regulon.

In addition, the expression of *sigE* was also dysregulated in the *whiB4*::Tn mutant during resuscitation. In WT M. marinum, the mRNA level of *sigE* was markedly reduced during reaeration (Fig. 4D), which is consistent with a previous finding in M. tuberculosis (23). In contrast, the expression of *sigE* continued to increase in the *whiB4*::Tn mutant under the same conditions (Fig. 4D). Taken together, the dysregulation of the DosR regulon and *sigE* in the *whiB4*::Tn mutant likely contributes to its defective resuscitation from the hypoxia-induced dormancy (Fig. 4B).

In summary, our *in vivo* zebrafish infection experiments and *in vitro* analysis of resuscitation suggest that WhiB4 is a major determinant of pathogenesis and disease progression of M. marinum infection in zebrafish. We suggest that WhiB4 is an attractive target for the development of novel therapeutics, which may help to prevent the reactivation of LTBI, thereby reducing the incidences of active TB. Future studies in this area are warranted.

## MATERIALS AND METHODS

### Bacterial strains and culture conditions.

The Mycobacterium marinum strain 1218R, the transposon insertion *whiB4::*Tn mutant of M. marinum, and the complemented strain were described previously (29). M. marinum cells were grown at 30°C in Middlebrook 7H9 broth or 7H10 agar (Difco) supplemented with 0.2% glycerol (7H9) or 0.5% glycerol (7H10), 0.05% Tween 80, and 10% oleic acid albumin dextrose catalase.

### Molecular cloning.

To generate a recombinant M. marinum expressing FLAG-tagged WhiB4 for the ChIP-seq analysis, the *whiB4* gene was amplified and a FLAG tag at C-terminal by PCR from M. marinum 1218R genome with the forward primer 5′-ACCGGATCCGTGTCGGGAATTCGTCCTGTTGA-3′ and the reverse primer 5′-CCCAAGCTTCTACTTATCGTCGTCATCCTTGTAATCTCCGACGCTGCGGCG-3′ was added. The resulting PCR product was digested with BamHI and HindIII restriction enzymes and ligated with the pMV261 vector predigested with the same enzymes. The resulting plasmid, pMV261-WhiB4, was transformed into M. marinum 1218R by electroporation, and transformants were selected on Middlebrook 7H10 agar containing kanamycin (25 μg/mL).

### Ethics statement.

All of the animal procedures were approved by the local animal care committees at Fudan University. All methods were performed in accordance with the relevant guidelines and regulations.

### Zebrafish infection.

Zebrafish infection with M. marinum was performed as described previously (45). Briefly, adult zebrafish (AB strain, 3 to 4 months old, 20 per group) were anesthetized in 0.1% tricaine and infected by intraperitoneal injection of 10^4^ CFU bacteria per fish or PBS as the negative control and monitored for their survivals.

To determine the bacterial load, fish were sacrificed and incubated with 75% ethanol for 5 min to kill bacteria on the surface. They were then rinsed with sterile PBS and homogenized in sterile PBS using the NOVAprep DS1000 (NewZongKe) at 5,000 rpm for 3 20-s cycles with 300-s pauses. The homogenates were diluted and plated to determine the CFU of M. marinum.

For histopathological analysis, three fish were sacrificed at each time point. Fish were fixed in 10% phosphate-buffered formalin and then decalcified with 20% EDTA–citrate. After dehydration with ethanol, specimens were placed in xylene and embedded in paraffin; then, 5-μm serial paraffin sections were prepared and subjected to hematoxylin and eosin staining and Ziehl–Neelsen acid fast staining. The slides were observed under a Nikon Ni-U microscope, and images were collected with a digital camera.

For determination of the host immune response, kidney, the main hematopoietic organ of adult zebrafish, was isolated after 2 weeks of infection (46). The sacrificed zebrafish were dissected and the kidneys attached to the spinal column were removed. Twenty-five kidneys per group were collected and pooled as one sample. They were incubated with RNA-Be-Locker A reagent (Sangon Biotech) for 3 h at 4°C to stabilize RNA according to the manufacturer’s recommendations and kept at −80°C for RNA extraction. Three biological replicates were performed.

### Antibiotic treatment.

Two weeks after infection, zebrafish were exposed to antibiotics administered through a chronic bath treatment (47). The doses of antibiotics were optimized (ethambutol, 1.0 mM; rifampicin, 0.4 mM; moxifloxacin, 0.3 mM) following a previous study (48). Antibiotic stock solutions were prepared in dimethyl sulfoxide (DMSO) and diluted in seawater. The water was changed daily. Antibiotics were added each day to each treatment group, and an equal volume of DMSO as the solvent control was added to the control group. After 1 or 2 weeks of treatment, five fish in each group were taken to determine the bacterial load.

### *In vitro* hypoxia and reaeration treatment.

Cultures of M. marinum were grown to mid-log phase (optical density at 600 nm [OD_600_] of 0.4 to 0.45) and then transferred to conical flasks with a headspace ratio of 0.5 (32). The flasks were sealed and incubated in the dark at 30°C with slow stirring. On day 8, the screw cap was replaced with a permeable membrane to introduce oxygen into the system. Samples were taken 0, 0.5, 4, 24, and 48 h after reaeration for CFU plating and RNA extraction.

### RNA sequencing.

For RNA-seq analysis of zebrafish kidney, a total of 6 samples were collected (three biological replicates, WT- and whiB4::Tn-infected animals) and analyzed.

For comparison of *whiB4*:Tn and WT, a total of 30 samples were collected (three biological replicates at each time point) and analyzed. For comparison of the complemented strain and WT, a total of 25 samples were collected (three biological replicates at each time point for the complemented strain and two biological replicates at each time point for WT) and analyzed.

Total RNA extraction, cDNA library construction, and Illumina sequencing were performed as described previously (29). Briefly, bacterial RNA was extracted using the Tianmo TR205-200 kit (Tianmo) according to the manufacturer’s protocol. For RNA extraction of zebrafish kidneys, the samples were removed from RNA-Be-Locker A reagent by centrifugation and added with 1 mL TRIzol for lysis. The insoluble tissues were removed by centrifugation, and supernatants were collected and added with equal volume ethanol. The RNA was then extracted using the Tianmo TR205-200 kit. The transcriptomic analysis and DEG identification were performed as described previously (49).

For RT-qPCR validation of zebrafish kidney RNA-seq results, 1 μg RNA sample extracted as described above was treated with DNase and reverse-transcribed following the manufacturer’s instructions using the PrimeScriptTMRT reagent kit with gDNA eraser (TaKaRa). The resulting cDNA was used as the template for PCR amplification using primers listed in Table S7. The expression of 18S rRNA was used as an internal control and for normalization of the host genes (50). The RT-PCRs were performed in 10 μL reaction mixtures containing 5 μL of 2× Talent qPCR PreMix, 0.2 μL of 50× ROX reference dye (TIANGEN), 0.3 μM forward and reverse primers, and 4.2 μL of cDNA template (1:100 diluted for 18S rRNA and 1:10 diluted for the rest of the genes). The RT-PCR was performed using the QuantStudio 7 real-time PCR system (Thermo Fisher). PCR was carried out at 95°C for 5 s and 60°C for 10 s, for a total of 40 cycles. The data were analyzed by ΔΔ*CT* method. The mRNA fold changes of target genes were calculated by normalizing against the 18S rRNA mRNA level and wild-type 1218R strain.

### GO and KEGG pathway analysis.

The gene ontology (GO) database (51) (www.geneontology.org/) and KEGG database (52) (www.genome.jp/kegg/) were used for data analysis. Chi-square test and Fisher’s exact test were performed to determine whether one set of genes was more enriched than another. Enrichment analysis was performed as described previously (49).

### ChIP-seq analysis.

For ChIP-seq, 50 mL M. marinum cultures grown to log phase (OD_600_ of 0.8) under normoxia, or 100 mL M. marinum cultures grown under hypoxia (7 days) or 0.5 h after reaeration, were crosslinked by 1% formaldehyde for 45 min at 30°C with slow stirring. Crosslinking was quenched by adding glycine (250 mM) and incubating for 15 min at 30°C with slow stirring. The bacteria were collected, washed with cold PBS, and resuspended in 0.5 mL of immunoprecipitation (IP) buffer (20 mM HEPES [pH 7.9], 50 mM KCl, 0.5 M dithiothreitol [DTT], 10% glycerol) containing the protease inhibitor cocktail (Roche). Samples were lysed by sonication for 15 min. An average size of 100- to 500-bp DNA fragments was observed by agarose gel electrophoresis. Then, cell debris was removed by centrifugation and the supernatant was retained. The salt concentration of the supernatant was adjusted to a final concentration of 10 mM Tris–HCl (pH 8.0), 150 mM NaCl, 0.1% NP-40 (IPP150 buffer). Immunoprecipitation of FLAG-tagged proteins was carried out by incubating the lysate with 50 μL of anti-FLAG M2 magnetic beads (Sigma) on a nutator at 4°C overnight. The beads were washed twice with IPP150 buffer followed by once with Tris-EDTA (TE) buffer. DNA from magnetic beads was eluted by incubation with 250 μL elution buffer (50 mM Tris-HCl [pH 7.5], 10 mM EDTA, 1% SDS) at 65°C for 40 min. Elution was treated with RNase A for 1 h at 37°C and decrosslinked by incubation with 1 mg/mL proteinase K at 50°C for 2 h and 65°C overnight. DNA was purified using phenol–chloroform extraction.

Three ChIP-seq analyses were performed, one from each sample at different time points (hypoxic cultures at 0 and 0.5 h postaeration and log-phage cultures grown under aerobic conditions). Sequencing was performed on the Illumina HiSeq platform. Coverage along the genome was calculated using Bowtie2 (53). Input genomic DNA without IP was used as a control, and the peaks were significantly enriched in ChIP samples compared with input genomic DNA, as determined by MACS2 (54) with a cutoff of enrichment fold of ≥1.5 and a *q* value of <0.05.

### Statistical analysis.

Statistical analysis was performed using the GraphPad Prism version 9.9.0.

### Data availability.

Raw data of RNA sequencing and ChIP sequencing can be retrieved using the GEO platform numbers GSE178191 and GSE189123.
