# Supplementary material for: WhiB4 Is Required for the Reactivation of Persistent Infection of Mycobacterium marinum in Zebrafish
Source: Microbiol Spectr. 2022 Mar 10;10(2):e00443-21. doi: 10.1128/spectrum.00443-21 (PMC9045381; doi:10.1128/spectrum.00443-21)
Supplement: SUPPLEMENTAL FILE 1 — Supplemental material. Download SPECTRUM00443-21_Supp_1_seq16.pdf, PDF file, 0.4 MB [file spectrum00443-21_supp_1_seq16.pdf]

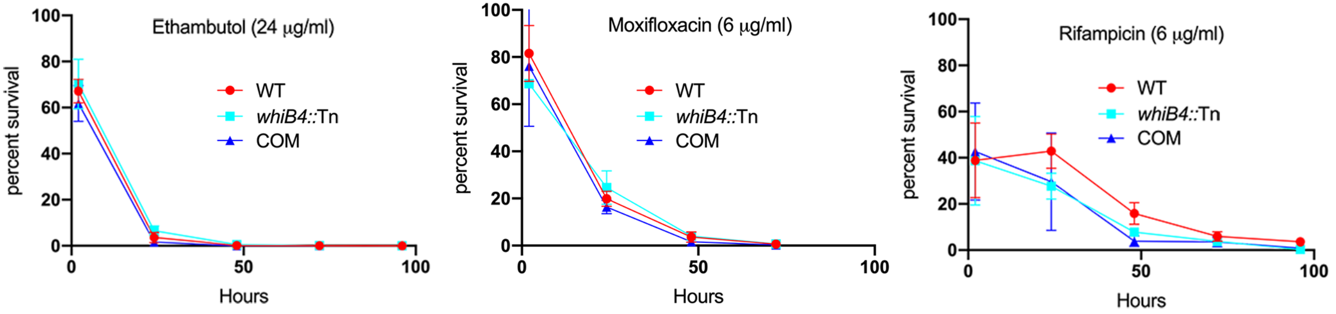

Ethambutol (24 µg/ml)
percent survival
100
80
60
40
20
0
0
50
100
Hours
WT
whiB4::Tn
COM
Moxifloxacin (6 µg/ml)
percent survival
100
80
60
40
20
0
0
50
100
Hours
WT
whiB4::Tn
COM
Rifampicin (6 µg/ml)
percent survival
100
80
60
40
20
0
0
50
100
Hours
WT
whiB4::Tn
COM

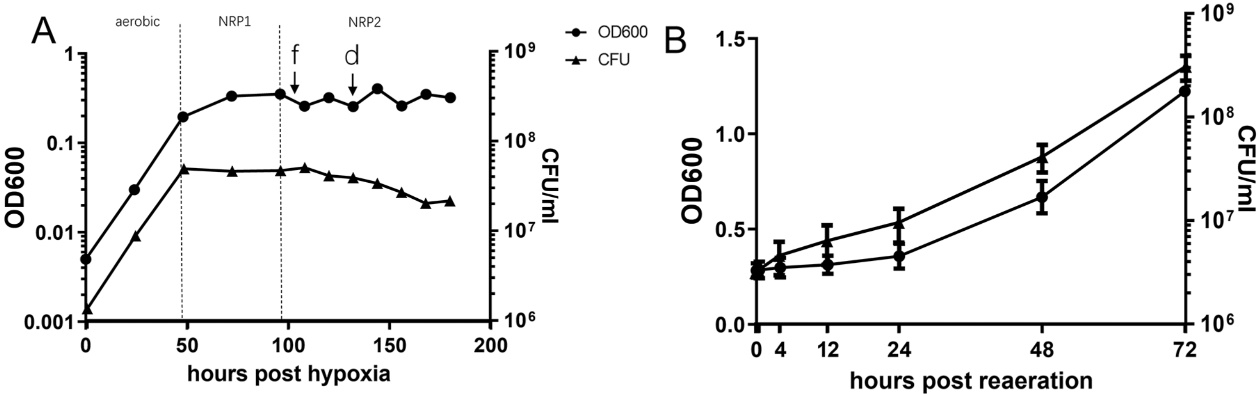
A
aerobic
NRP1
NRP2
f
d
OD600
CFU
1
0.1
0.01
0.001
10^9
10^8
10^7
10^6
CFU/ml
0
50
100
150
200
hours post hypoxia
B
1.5
1.0
0.5
0.0
0
4
12
24
48
72
hours post reaeration

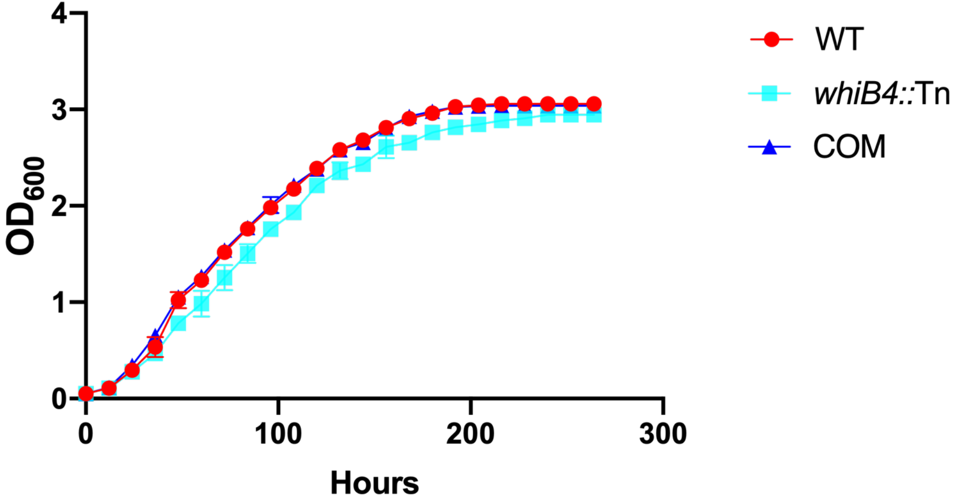

OD600
Hours
0
1
2
3
4
0
100
200
300
WT
whiB4::Tn
COM

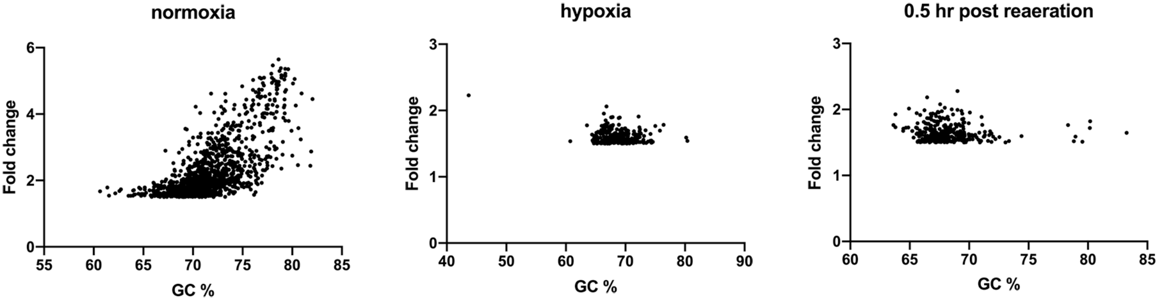
normoxia
hypoxia
0.5 hr post reaeration
Fold change
GC %
0
2
4
6
55
60
65
70
75
80
85
0
1
2
3
40
50
60
70
80
90
0
1
2
3
60
65
70
75
80
85

Supplementary Information

Figure S1. *In vitro* drug tolerance assay. The MIC of ethambutol, moxifloxacin, and rifampicin against WT, *whiB4*::Tn and the complemented strain were the same, which were 8-, 2-, and 2-µg/ml, respectively. Equal number (5×10^3 CFU/ml) of bacterial strains were treated with 3×MIC of each drug, and at the indicated time points, aliquots were removed and plated for CFU counts. The percent survival was calculated by dividing the CFU of drug treated cultures by the CFU of the same cultures untreated but were incubated for the same period. Data are from three biological replicates. There were no statistical differences on the time-to-kill curves of the three strains for each drug, as analyzed by two-way ANNOVA.

Figure S2. The Wayne model of *M. marinum* growth under hypoxia and upon reaeration. (**A**) A sealed and slowly shaking culture was incubated over an extended period where the bacteria deplete available oxygen. The gradual depletion of oxygen leads to nonreplicating persistence states (NRP) with a concomitant shift in gene expression and metabolism. Under these conditions, *M. marinum* entered the NRP 1 state after 48 hr incubation, characterized by a slight increase in turbidity without a corresponding increase in CFU. Full anaerobiosis was achieved after 132 hr incubation, as indicated by the complete decolorization of methylene blue, and the bacteria were in the NRP2 state. (**B**) Growth curve of *M. marinum* hypoxic culture (7-day incubation under hypoxia) upon reaeration.

Figure S3. Growth of the WT, *whiB4*::Tn and the complemented strain (COM) under aerobic conditions (normoxia). The bacteria were grown in 7H9 media under aerobic conditions and the data (mean ± SD) are from three biological replicates.

Figure S4. **ChIP-seq analysis of WhiB4 binding sites in the genome of *M. marinum*.** The fold-change of binding sites were plotted against the percentage of GC content in the sequences.

Table S1. List of differentially expressed genes (DEGs, fold difference >1.5, *p*<0.05) detected between zebrafish infected with *whiB4*::Tn and WT.

Table S2. GO analysis of DEGs between zebrafish infected with *whiB4*::Tn and WT.

Table S3. KEGG analysis of DEGs between zebrafish infected with *whiB4*::Tn and WT.

Table S4. RNA-seq reads of WT, *whiB4*::Tn and the complemented strain grown under different conditions.

Table S5. RNA-seq analysis of DEGs between *whiB4*::Tn and WT grown under different conditions. The *pe/ppe* family genes are highlighted.

Table S6. RNA-seq analysis of DEGs between the complemented strain (COM) and WT grown under different conditions.

Table S7. ChIP-seq analysis of WhiB4 binding sites in WT grown under different conditions. Genes that are at the *dosR-dosS* locus are highlighted.

Table S8. Primers for RT-qPCR of host genes.

Table S8. Primers for RT-qPCR

primers	5’-3’
18S rRNA F	TCGCTAGTTGGCATCGTTTATG
18S rRNA R	CGGAGGTTCGAAGACGATCA
TGFbr1bF	GGTGTGTGTGCTGTGTTTCC
TGFbr1bR	TCCAGATGTGGTCATGTCGT
TGFb1aF	AACTACTGCATGGGGTCCTG
TGFb1aR	GGACAATTGCTCCACCTTGT
IL2rgaF	ATGGCTGTCCATGCCTAAAG
IL2rgaR	AGCAGATCCGGGTATTGTTG
IL6rF	ACGGCCAGTGTGTTACCTTC

IL6rR	CTGGCCTTAGGGTGTCATGT
IL10F	ATTTGTGGAGGGCTTTCCTT
IL10R	AGAGCTGTTGGCAGAATGGT
IL17ra1aF	GCGAGAGTAGAAACGGATGC
IL17ra1aR	TCGGGCGTCTCATTAATTTC
IL6stF	CTGGTCAGTGAACGCGACTA
IL6stR	AAGACGCTCAGGAACAGGAA
IL2rgbF	ATCTGGCAACGGAACTTGTC
IL2rgbR	GGTGGGTTTAGCAGCACATT
IL12rb2F	AGCAAAGGAAGCAGAAACCA
IL12rb2R	CATTTATTCGGCGTGGAACT
CXCL8aF	GTCGCTGCATTGAAACAGAA
CXCL 8aR	CTTAACCCATGGAGCAGAGG
IL2rbF	GATGAGCAGCATTGTGAGGA
IL2rbR	TCTGCTGGCTCACAGAGAGA
TNFaF	GCTTATGAGCCATGCAGTGA
TNFaR	TGCCCAGTCTGTCTCCTTCT
CXCL8b.3F	ACTGCCCATCCAGCAGTTAC
CXCL8b.3R	TCCTGCCTTCGATGATCTTT
IL17rcF	TGCTTTCTGAGCTGGGTTTT
IL17rcR	TGTCTTTCCACTGCAGCATC
IFNgr1lF	GGGGATCCAGTTGGGACTAT
IFNgr1lR	GAGGTTTTCAGCGTCTGAGG
TGFb1bF	CTAGACCCACTGCCCATCAT
TGFb1bR	GAAAGTGCTGGGTCTCAAGC
mmp9F	GCCCTGATCGTGGATACAGT
mmp9R	AGGGCCAGTTCTAGGTCCAT
mmp13aF	GACCAAGACACACTCGCAGA
mmp13aR	GCTTTCCAGTCACCTTGAGC

42
